# Interventions for the control of Crimean-Congo hemorrhagic fever and tick vectors

**DOI:** 10.1038/s41541-024-00970-5

**Published:** 2024-10-01

**Authors:** José de la Fuente, Srikant Ghosh, Laetitia Lempereur, Aura Garrison, Hein Sprong, Cesar Lopez-Camacho, Christine Maritz-Olivier, Marinela Contreras, Alberto Moraga-Fernández, Dennis A. Bente

**Affiliations:** 1 SaBio, Instituto de Investigación en Recursos Cinegéticos IREC-CSIC-UCLM-JCCM, 13005 Ciudad Real, Spain; 2 Department of Veterinary Pathobiology, Center for Veterinary Health Sciences, Oklahoma State University, Stillwater, OK 74078 USA; 3 Entomology Laboratory, Parasitology Division, ICAR-Indian Veterinary Research Institute, Bareilly, 243122 Uttar Pradesh India; 4 Eastern Regional Station, Indian Veterinary Research Institute, Kolkata, 700037 West Bengal India; 5 One Health & Disease Control Group (NSAH-CJW), Food and Agriculture Organization of the United Nations, 00153 Rome, Italy; 6 Virology Division, US Army Medical Research Institute of Infectious Diseases, Frederick, MD 21702 USA; 7 Centre for Infectious Disease Control (CIb), National Institute of Public Health and Environment (RIVM), 3720 MA Bilthoven, The Netherlands; 8 The Jenner Institute, University of Oxford, Oxford, OX3 7DQ United Kingdom; 9 Department of Biochemistry, Genetics and Microbiology, Faculty of Natural and Agricultural Sciences, University of Pretoria, Pretoria, Gauteng South Africa; 10 Galveston National Laboratory, Institute for Human Infection and Immunity, Department of Microbiology & Immunology, University of Texas Medical Branch, Galveston, TX USA

**Keywords:** Viral infection, Conjugate vaccines

## Abstract

Crimean-Congo hemorrhagic fever (CCHF) is a zoonotic disease associated with its principal tick vector, *Hyalomma* spp. with increasing fatal incidence worldwide. Accordingly, CCHF is a World Health Organization-prioritized disease with the absence of effective preventive interventions and approved vaccines or effective treatments. This perspective raised from a multidisciplinary gap analysis considering a One Health approach beneficial for human and animal health and the environment exploring international collaborations, gaps and recommendations.

## Introduction

Crimean-Congo hemorrhagic fever (CCHF) is a zoonotic disease with a high fatality rate caused by the tick-borne CCHF virus (CCHFV)^1–3^. Isolated human cases and sometimes local outbreaks have been reported throughout Africa, the Middle East, Asia and southern and eastern Europe, and associated with principal tick vectors, *Hyalomma* spp. These ticks also cause substantial economic losses and health concerns in livestock with detrimental consequences for the environment. The CCHFV is expected to become endemic in new geographies with rise in disease incidence, vector distribution extent and local abundances in response to climate changes, environment and human (inter)actions. Accordingly, CCHF is included in the World Health Organization’s list of prioritized diseases due to its high fatality rate, global distribution, and the absence of an approved vaccine or effective treatment (https://www.who.int/activities/prioritizing-diseases-for-research-and-development-in-emergency-contexts; accessed December 19, 2023).

**Fig. 1:**
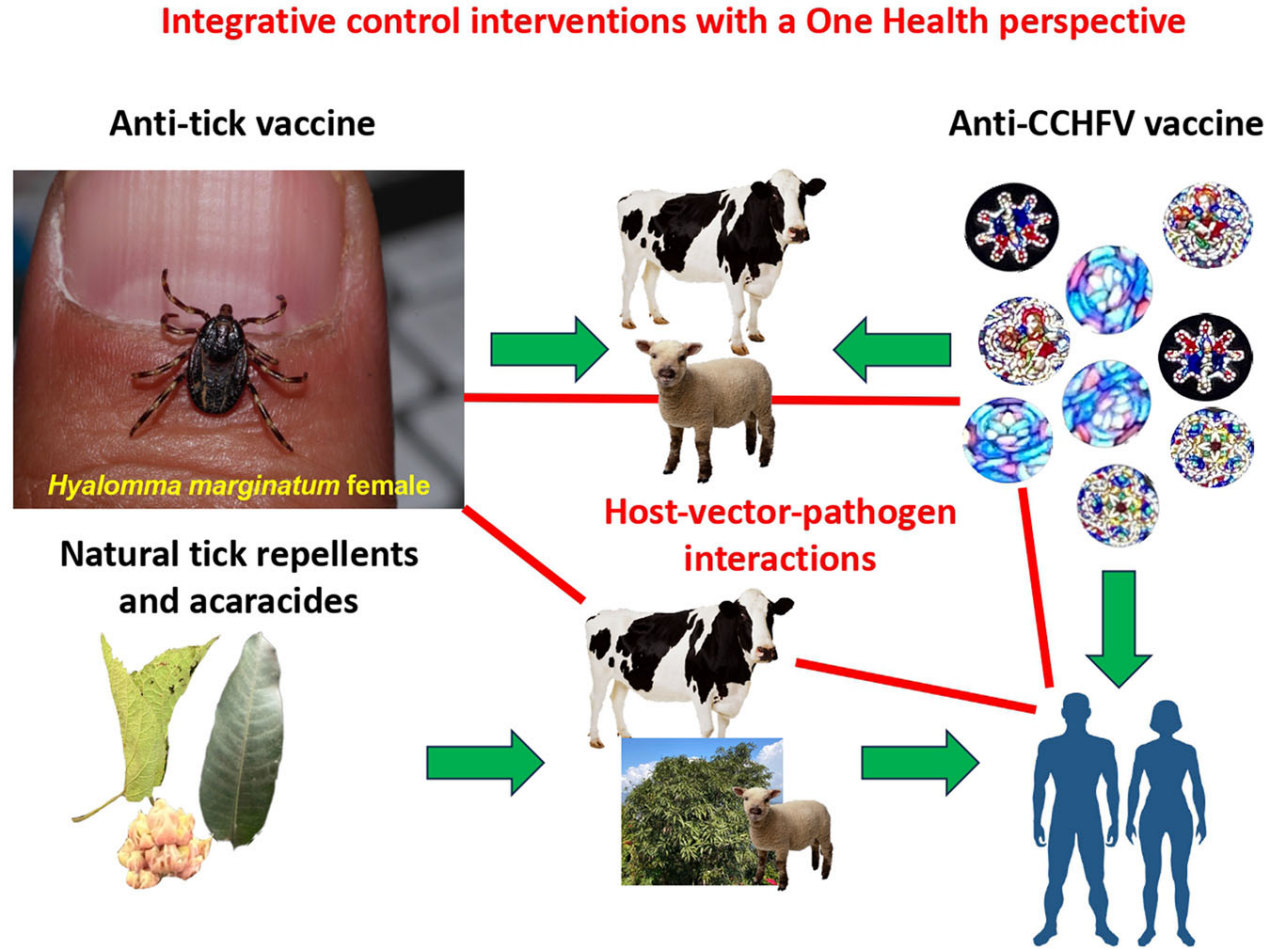
Proposed interventions for the control of CCHFV and tick vectors. A One Health approach for the control of CCHF targeting host-vector-pathogen interactions with integrative control interventions, including anti-tick vaccines for animal hosts, anti-virus vaccines for animal hosts and humans, and natural tick repellent and acaricide-derived products applied to both humans and animals. Images courtesy of co-authors H. Sprong (tick) and J. de la Fuente (plant leaves, cow, sheep and virus representations). Human figures were adapted from free drawings available at https://www.vecteezy.com/free-vector/male-female-body.

However, despite growing risks associated with CCHF, bibliometric analysis (Supplementary Fig. 1) highlights how publications related to CCHF vaccines increased since 2015 but still with no more than 30 papers per year. To approach this challenge, a multidisciplinary CCHF gap analysis workshop was organized at Montpellier, France, on November 28–30, 2023. A One Health approach is proposed, which aims to be beneficial for human and animal health and the environment (Fig. 1). Furthermore, we explore how international collaborations can help to prevent local/regional disease outbreaks and how local efforts can help to prevent further spread of the disease.

## Epidemiology

The increasing health threats caused by CCHFV can be considered as a tip of the iceberg^4^. Isolated human cases and sometimes local outbreaks have been reported throughout Africa, the Middle East, Asia, and southern and eastern Europe, including Crimea, Astrakhan, Rostov, Uzbekistan, Kazakhstan, Tajikistan, Democratic Republic of the Congo, Uganda, Mauritania, Iraq, the United Arab Emirates, Saudi Arabia, Pakistan, Iran, Bulgaria, Turkey, Russia, Spain, and India, and associated with principal tick vectors, *Hyalomma* spp. (*H. marginatum*, *H. anatolicum*, *H. asiaticum*, *H. dromedarii*, *H. rufipes*, *H. truncatum*, *H. turanicum*, and *H. impeltatum*)^3,5–21^.

In most endemic areas in Africa and Asia, livestock herders and workers, butchers, and slaughterhouse workers are at the highest risk of CCHFç^22–24^ Transmission to these occupational groups not only occurs through bites from infected ticks, but mostly through direct contact with infected blood, secretions, tissues, or engorged ticks from viremic animals^24^. Most domestic animals can be viremic without presenting any symptoms^25,26^, making it difficult to discern infectious from non-infectious animals. Most livestock species act as propagation hosts for *Hyalomma* ticks, which are well adapted to arid environment. Therefore, the higher occurrence of livestock grazing in semi-natural grasslands with *Hyalomma* ticks with higher abundances gives rise to a higher risk of exposure to CCHFV. Remarkably, a far bigger concern of livestock farmers in those areas is production loss through infestation by *Hyalomma* and other tick species and tick-borne diseases (TBD) such as piroplasmosis. For control, acaricides are extensively used and often without maintaining standard guidelines^27^. The widespread exposure to acaricides, often at sub-effective concentrations, has selected resistance in many tick species feeding on livestock. The development of resistance is particularly significant in single-host cattle ticks, but resistance is also reported in *Hyalomma* ticks throughout sub-Saharan Africa and parts of Asia. As a result, the livestock sector is confronted by an upsurge of ticks as well as TBD in areas where resistant ticks occur. Acaricide resistance is a global health problem as it affects animal, environmental and human health. Acaricide resistance can compromise human health by increasing the risks for food security (production loss), food safety (toxic residues in animal products) and the increased risk of TBD, most notably CCHF. To this end, control One Health approaches include human and animal CCHF-vaccine targeting the virus, and anti-tick vaccines to control multiple TBD affecting both human and animal health worldwide^14,28^. The prevention and control of CCHF should include the innovation in integrated management involving improvement of practices for livestock grazing in semi-natural grasslands, and alternative tick control methods such as anti-tick vaccines and tick repellents/phytoacaricides for the control of multiple tick species and transmitted pathogens.

## Mechanisms/pathophysiology

Ranchers, shepherds, butchers, farmers and abattoir workers are most vulnerable to CCHF, but some cases have also been reported in medical and nursing personnel due to nosocomial transmission. Highest CCHF fatality rates were reported in agricultural (28.9%), healthcare (19.2%), slaughterhouse (16.7%) workers, and farmers (13.9%)^1^. The average mortality rate is 10–40%, but it might vary from as low as 0–5% in Iran to as high as 60–80% in different regions^2^.

Reviewing the CCHF cases reported from different countries, it is observed that the most common complication of the disease has been hematologic disorders, of which thrombocytopenia and increased Partial Thromboplastin Time and Prothrombin time are the most common disorders seen in 100% of CCHF patients^1,2^. Bleeding in different organs, especially the oral cavity, is the next common complication^1^. The usual causes of death are shock, disseminated intravascular coagulation (DIC), and multi-organ failure, including hepatic, renal, and respiratory failure. Moreover, rare complications such as intracerebral hemorrhage, compartment syndrome, intra-abdominal, pleural and pericardial effusions, acute pancreatitis, myocarditis, and cholecystitis are reported.

Treatment options are mainly based on enhanced post-exposure prophylaxis, including molecular and serological tests, ribavirin, and supportive treatment, which should be readily available and promptly accessible to HCWs at risk of exposure. In human pathogenesis, the role of adaptive immune responses against CCHFV is not clear but non-neutralizing antibodies were reported to be protective against lethal CCHFV challenge, demonstrating that antibodies can be protective via mechanisms other than neutralization^28^.

## Diagnosis, screening and prevention

Research on human anti-CCHFV vaccines has advanced in the last years, with chimpanzee adenoviral vector with M-segment (ChAdOx2 CCHF)^29^ and Modified Vaccinia Ankara (MVA)-CCHF (https://www.hra.nhs.uk/planning-and-improving-research/application-summaries/research-summaries/phase-i-vaccine-study-of-mva-cchf/) vaccines under phase 1 clinical trials (Supplementary Table 1). Recent developments in mRNA vaccine research have provided new platforms for human CCHF control, including nucleoside-modified mRNA vaccines that have shown promise in protecting IFNAR−/− mice against CCHFV infection, vaccines using lipid nanoparticles to deliver mRNA encoding CCHFV nucleoprotein or glycoproteins with strong humoral and cellular immune responses in both IFNAR−/− and immunocompetent mice^30^ (Table 1). Animal anti-CCHFV vaccines have also been considered to provide an additional intervention for the control of CCHF^31^. However, from a One Health and/or veterinary/animal perspective, we may question the potential impact of the animal anti-CCHFV vaccines (Table 1). Additional information and references are in Supplementary Table 1.

**Table 1:** Considerations for human and animal anti-CCHFV vaccines

Human anti-CCHFV vaccines	
1	Vaccine efficacy studies require mice lacking type 1 interferon signaling, either knockout mice or the use of anti-IFN-I antibody. The very short disease window is quite stringent for successful intervention.
2	While mRNA vaccines show promise, their effectiveness in humans, long-term immunity, and scalability for widespread use need further research. It is important that big pharmaceutical companies invest to advance the pre and clinical development.
3	The use of MAR-5A3 (anti-IFN-I antibody) allows for both protective mechanism and pathogenesis studies in any knockout mouse model system, which may be less stringent than IFNAR−/− or STAT1−/− mice. These studies are needed as the correlates of protective efficacy for the majority of the pre-clinical vaccines are unknown, and neutralizing antibody responses do not correlate.
4	Both glycoprotein and nucleocapsid protein-based vaccines are effective, but this may be dependent on vaccine format. Is the inclusion of both advantageous for optimal cross-protective efficacy against diverse isolates?
5	Although some studies have only tested homologous challenge, heterologous challenge has demonstrated it is possible for broadly protective vaccines. Nevertheless, overall ecological impact of all human activities must be considered for human and animal vaccines.
6	The non-human primate (NHP, cynomolgus macaque) model is primarily a disease model with several measurements that can be used for human vaccine and therapeutic studies to identify protective countermeasures (viremia, temperature, cytokines). Lethal disease is not reproducible beyond first publication and successful intervention criteria is not well defined. NHPs do not recapitulate severe human disease. The most robust challenge route requires intravenous infection and the mild disease endpoints, other than viremia, are not consistent.
7	Unlike mice, minimal to no neutralizing response to glycoprotein-based vaccines in NHPs, DNA and RNA-based vaccines have been tested to date in NHPs. Although neutralizing antibodies are not a direct correlate, the divergent response in the mouse and NHP model is notable.
8	Bulgarian CCHFV vaccine is the only vaccine used and minimally studied in humans (in Bulgaria only) with some measurable neutralizing antibody activity and T-cell responses to NP. Animal studies are lacking with this vaccine for comparison with other pre-clinical vaccines in development.
9	Ideally advanced vaccine studies should include determining if there is full clearance of virus up to day 28 post-infection in the cynomolgus macaque model. Viral persistence has been demonstrated in numerous tissues in some NHP animals (including testes), and there is some evidence of persistence in humans. Persistence appears to be a common theme for hemorrhagic fever viruses.
10	Are NHP studies needed for licensing human vaccines for CCHFV? Several pre-clinical vaccines have gone to human trials (Phase I and Phase II) without NHP efficacy data (e.g., HTNV DNA, CCHFV ChAdOx2 CCHF). SARS-CoV-2 vaccines were tested in humans (emergency use) without NHP data. However, since NHPs are showing divergent responses compared to mice, NHP work is justified to better predict immunogenicity and efficacy in humans and avoid trials of vaccines that likely will be non-immunogenic in humans.
11	Lack of another immunocompetent model may require the use of NHPs. However, MAR-5A3 mouse model is immunocompetent at the time of vaccination and only requires type I IFN blockade at the time of challenge.
12	What population should receive a vaccine? Only higher-risk individuals in endemic areas such as HCW, butchers, abattoir workers, farmers, hunters? Cases are sporadic in endemic areas.
**Animal anti-CCHFV vaccines**	
1	Domesticated animals do not suffer from CCHF-infections; hence, there is no incentive for farmers to give/pay for that vaccine.
2	Humans live longer than livestock, also the vaccination of human risk groups can be much more targeted than that of livestock, therefore it is much wiser/effective.
3	Such a vaccine most likely will not break the transmission cycle as wildlife (such as hares and rodents) can also be infected with the virus. Therefore, infected ticks can still bite the cattle, and farmers might still get infected by crushing the tick.
4	A CCHF vaccine will not “solve” the tick problem on livestock, and it doesn’t solve the issues with acaricides.
5	An animal anti-CCHFV vaccine may have ecological implications with negative impact due to increase in CO_2_ greenhouse gas due to vaccine packaging, storage and deep-freezing.
6	Potential alternative to consider: A Point of Care CCHFV-antigen test for cattle/hare for slaughterhouses/butchers? If available should this be only used for epidemics?

Currently, the control of ticks infesting livestock depends on commercial synthetic chemical acaricides. Seven different classes of synthetic chemical acaricide products against ticks are currently in the market for use on livestock: (1) Organophosphates (e.g., Diazinon), (2) Carbamates (e.g., Carbaryl), (3) Formamidines (e.g., Amitraz), (4) Phenylpyrazoles (e.g., Fipronil), (5) Pyrethroids (e.g., Permethrin, Flumethrin, Deltamethrin, Cypermethrin), (6) Macrocyclic Lactones (e.g., Ivermectin, Moxidectin), and (7) Isoxazoline (e.g., Fluralaner) being newly developed and with a current limited access.

These products are subject to marketing authorizations that depend on the country’s regulations. Its distribution and accessibility also vary according to the market and the financial capacity of farmers. These products are available in various formulations (e.g., pour-on, injectable, spray) and generally cover a broader spectrum of arthropod control with acaricide or insecticide indications as well as with anthelmintic activity (macrocyclic lactones), which in some cases make it more difficult to develop and implement for tick control measures.

The widespread exposure to acaricides, often at sub-effective concentrations, has selected resistant strains from tick populations^32,33^. The ability of ticks to develop resistance against different classes of acaricides is aggravated by malpractices in the applications, the use of substandard products, and the lack of strategies to delay selection for resistance. The development of resistance is particularly significant in single-host cattle ticks throughout vast areas in sub-Saharan Africa, Latin America, and parts of Asia. Moreover, resistance is also reported in multi-host tick species such as *Rhipicephalus appendiculatus*^34^ and *Hyalomma* spp^35–37^. As a result, the livestock sector is confronted by an upsurge of TBD in areas where resistant ticks occur.

Resistance in cattle ticks has been reported against all acaricidal classes (organophosphates, synthetic pyrethroids, formamidines and macrocyclic lactones), leading to significant economic losses for cattle producers globally. In addition to animal welfare, health risks and production losses, food safety with potential residues, pathogen transmission to humans, exposure to toxicity, and pollution affecting ecosystems health and biodiversity are other potential associated negative effects. The current situation remains critical, without sustainable pasture-grazed livestock in some regions. Therefore, from health and ecological perspectives, new considerations should be considered (Table 2).

**Table 2:** Future perspectives and considerations to acaricides

1	Alongside the need for regulation, policy, empowerment and awareness of stakeholders and the allocation of resources, practical and innovative solutions are required for the sustainable management of ticks and TBD in livestock for enhanced food security and healthier livestock as well as reduction of risk of zoonotic diseases.
2	In the short term, data production, collection and sharing of data on ticks, TBD and acaricide resistance will be needed to provide a better understanding of the situation, assessment of risks and a better response. Advanced mathematical models will also be needed with a combination of data aggregation and prediction of future events in a meaningful surveillance system.
3	Improvement in understanding of acaricide resistance mechanisms and diagnostic methods, including bioassays and molecular tools, will be urgently needed.
4	Intervention strategies will also have to be proposed in order to mitigate acaricide resistance in the first instance while promoting the proper use of acaricides.
5	Collective development of economically, socially, and environmentally sustainable interventions will be further needed and be gathered in an integrated tick management approach. These innovations should be efficient, safe, field-validated, cost-effective and environmentally sustainable. They should cover both herd management (biosecurity, nutrition, cattle genetics, pasture management) and alternative tick control methods (phytoacaricide, anti-tick vaccine, among others). Combining these complementary solutions should be evaluated at the field level, considering the sustainability and the cost-effectiveness.
6	Integration of educational and behavioral sciences will be needed to understand barriers implementation and shape interventions taking into consideration sociological drivers such as cultural, structural, psychological, gender and economic to acknowledge the complex and diverse factors that shape human behavior.
7	Natural acaricides and repellents do not affect the environment and may be used for integrated tick management by combining acaricides with host genetics, anti-tick vaccine and pasture management. Therefore, to mitigate the problem of acaricide resistance in an environmentally safe manner, the possibility of development and use of phyto-formulations is targeted by many researchers. A good number of plant extracts have been tested against different tick species with significant efficacy (Supplementary Table 2). However, the in vitro generated data have not been validated in vivo in most cases.
8	A good number of extracts/EOs prepared from different plant parts were tested in vitro with significant effects on all the stages of the tick vector (Supplementary Table 2).
9	A significant progress has been made in the development of green formulation effective against multi-acaricide-resistant ticks infesting animals. Technology is developed and ready for commercialization subject to regulations.

Additionally, it is important to consider alternative approaches to acaricides. In livestock, field burning^38^ is not possible or desirable everywhere as it is not environmentally friendly and unsustainable. Zero grazing or modern indoor housing is also not possible, feasible or desirable everywhere. Host resistance has been considered as a long-term solution^39^. From a nature environment perspective, culling tick wild hosts has been proposed but without evidence of effectiveness and the risk to biodiversity and ecosystem functioning^40^. Furthermore, livestock may be the major tick host associated with the risk of disease transmission to humans. Other biological methods with poor or no evidence of field effectiveness include natural tick enemies (bird/parasitic wasp) and entomopathogenic fungi/nematodes with possible side effects.

Several anti-*Hyalomma* spp. vaccine candidates have been identified (Supplementary Table 3). Vaccine protective antigens evolved from extracts of larvae and nymphs^41^ to purified antigens^42^ to recombinant Bm86 homologs from different *Hyalomma* spp^43–46^. and other recombinant antigens such as Subolesin (SUB), Calreticulin (CRT), Cathepsin-L like cysteine protease (CathL), Ferritin 2 (FER2) and Tropomyosin (TPM) ortholog of *H. anatolicum* were tested with 65.4%, 63.7%, 51.7%, 41.3% and 30.2% vaccine efficacy, respectively^47,48^ (Supplementary Panel 1).

In an attempt to develop cross-protective vaccines, Rodriguez-Valle et al.^49^ immunized cattle and camels with rBm86-based vaccine and challenged with *H. dromedarii* and *A. cajennense* with partial or no efficacy against these tick species. Similarly, cross-protective efficacy of Gavac™ was assessed against *H. anatolicum* with Indian tick strain-derived Bm86 antigen with 25% efficacy^50^. Recently, Nandi et al.^51^ synthesized two multi-epitope peptides (MEPs) and used it for immunization of rabbits against *H. anatolicum* infestations with more than 80% protection against challenge larvae and adults.

Despite these results in anti-*Hyalomma* vaccine research, additional considerations to advance in anti-tick vaccines are required (Table 3).

**Table 3:** Considerations to advance in anti-*Hyalomma* tick vaccines

Considerations	References	
1	The identification of tick protective antigens as vaccine candidates for the control of tick infestations and pathogen infection/transmission have advanced in the last 30 years. However, why new vaccines for tick control have not been registered and commercialized since release of Bm86-based vaccines Gavac and TickGard in the early 90s?	^62–66^
2	For ticks like *Hyalomma* spp., reservoirs and vectors of CCHFV that are promiscuous with a wide choice of hosts and have extended life cycles with each life stage is taking a season or more, the effectiveness of anti-tick vaccines remains to be demonstrated under field conditions. Vaccines that inhibit tick feeding and reproduction and/or reduce pathogen transmission will be most useful for *Hyalomma* spp.	^51,67^
3	A wide range of vaccine candidates have been and continue to be identified for the control of *Hyalomma* spp. (Supplementary Table 3). However, there is a need to down-select the best candidates for *Hyalomma* spp. to test using in vitro models and run proof-of-concept controlled field trials using relevant host species.	
4	Co-immunization protocol targeting different physiological processes of the vector has been proved effective. However, experimental data is required to identify the best combination of targets to be combined in multi-component vaccine to achieve the target of vector control with transmission-blocking properties.	^68^
5	New vaccine platforms such as quantum vaccinomics, virus-like particles and nanoparticles, oral formulations, and mRNA delivery platforms should be considered.	^64,69–77^
6	Innovative approaches are required that may be combined with vaccination such as autocidal tick control, paratransgenic ticks using RNA interference, transgenic symbiotic bacteria or *CRISPR*/*Cas9* technology with reduced fitness and/or vector competence, targeting immune response to tick midgut microbiota and glycoproteins containing glycan alpha-Gal, combination of tick and pathogen-derived protective antigens, genetic engineering of vector-borne obligate intracellular bacteria, and sterile tick technique among other.	^78–83^
7	Are wildlife vaccines required or practical? If so, oral formulations will be needed, and regular booster doses won’t be feasible. Note that for exposed antigens (like salivary antigens), hosts will be naturally challenged through biting, but will this help to reduce the number of vaccine boosters needed?	^84^
8	Combination of vaccines with botanical acaricides and biological control interventions.	^67,85,86^
9	What is the economic model/value proposition for implementing animal CCHF or anti-tick vaccines? There are lessons to be learned from the commercialization of TickGard and Gavac vaccines. Needs to be sustainable and resilient to commercial changes in company structures. Modeling tick vaccines is required to improve protective efficacy. These measures are also required for other tick control measures and infection treatment methods.	^87^
10	International collaborations regarding tick control by vaccination and technology transfer should be a must but currently lack the active involvement of funding agencies in middle-income countries, considering that the largest economic losses due to ticks on livestock occur in low- or middle-income countries.	^65^
11	Personalized vaccines are needed. Field trials with Bm86 and Subolesin vaccines evidenced that universal vaccines against tick infestations in different regions are not feasible. Vaccine antigens should be designed based on genetic information from local tick species.	^88^
12	Antigen combinations. An additional antigen that improved vaccine efficacy was not added to the Gavac/TickGard vaccines for commercial reasons. Furthermore, antigen combinations may reduce vaccine efficacy due to protein-protein interactions that reduce exposure of protective epitopes. The design of chimeric antigens with protective epitopes using quantum vaccinomics approached may contribute facing this challenge.	^69,70^
	Vaccine doses for effective and efficacious applications. TickGard required 3–4 boosters a year, not feasible in Australia where animals are not rounded up this frequently. However, GavacPLUS with Bm95 antigen was effective with one booster dose a year^89^. New adjuvants and membrane-exposed antigens and new vaccine platforms described above may improve vaccine efficacy while reducing number of doses.	^84,89–91^
	Self-disseminating vaccines should be considered to suppress zoonosis.	^92^
	Characterization of the immune protective mechanisms in response to vaccination.	^93^
	Immunization with a single antigen vaccine formulation may not be effective to protect animals from all the stages of the vector tick. Therefore, multi-antigen immunization protocols may be required.	

## Management

For the rational combination of different control interventions, insights come from lessons learned from other tick species. Different tick species such as *Argas reflexus*, *H. anatolicum*, *H. detritum*, *H. marginatum marginatum*, *Rhipicephalus sanguineus* and *Haemaphysalis* spp. have been identified as infected with CCHFV. Although these ticks are not all involved in disease transmission, it does pose a challenge to control viral load in hosts. The impact of the latter factor needs to be studied, as data remains limited regarding the role of various tick reservoirs and the maintenance of viral loads in hosts.

Regarding control of CCHFV and *Hyalomma* ticks, sensitivity analysis conducted by Lule et al.^52^ indicated that decreasing the tick survival time by (1) interfering with tick-to-tick transmission through co-feeding, and (2) reduction of CCHFV circulation through transstadial and transovarial transmission remains key points to lessen the disease cycle. Accordingly, interventions can be combined to control and/or reduce the risks of CCHF^53–55^ (Table 4). Additionally, challenges, limitations and future needs should be considered for successful implementation of integrated tick control strategies (Table 5).

**Table 4:** Combination of interventions to control and/or reduce the risks of CCHF

1	Reduction of immature tick loads. The nymphal and larval stages of *Hyalomma* spp. exhibit a preference for feeding on various hosts, including scrub hares, ground-frequenting birds (although birds generally display resistance to CCHF, with ostriches being an exception), gerbilline rodents, African hedgehogs, and multimammate rats. Notably, many of these animals are recognized reservoirs of the virus, adding complexity to tick control efforts. The broad spectrum of wildlife hosts presents a formidable challenge in devising effective tick control strategies. However, drawing inspiration from successful approaches employed in the mitigation of Lyme disease in the United States and Canada, valuable lessons have been learned on reducing tick-human contact and tick population sizes with multiple strategies for suppressing tick populations in rodents (Supplementary Table 4). Limited strategies for tick control on other small animal hosts are available.
2	Human and mature tick contact. Adult ticks feed on cattle, sheep, goats, horses, large wild herbivores (e.g., giraffes, eland, camels) and occasionally on dogs. These animals could be targeted by tick vaccines in the future, but current strategies may be applied to reduce human-tick contact and risks of infection (Supplementary Table 5).
3	Non-tick mediated infection. Crimean-Congo Hemorrhagic Fever also spreads through contact with infected tissues or blood from viremic animals, and human-to-human transmission. This risk is particularly notable when people handle, butcher, or consume infected livestock, especially ruminants and ostriches. Outbreaks have occurred in settings such as abattoirs, where workers are exposed to infected human or animal blood and contaminated items. Human-to-human transmission also takes place in clinical facilities, often involving infected blood and unsterilized medical instruments. Currently, PPE stands out as the most effective control strategy to mitigate CCHF transmission in abattoirs. However, the implementation of PPE can be challenging due to its cost and the necessity for strict adherence to proper usage, which is not consistently observed. Some information regarding the prevalence of CCHFV in slaughterhouses and the high-risk behavior of individuals working in these environments has been published.

**Table 5:** Challenges, limitations and future needs for successful integrated tick control strategies

Challenges	Limitations and future needs	References
Lack of public funding for tick control	Unlike mosquito control, suppression of tick populations remains the responsibility of individuals. In low and middle-income countries (LMIC) this is not feasible. In the USA, most homeowners are willing to pay $100–150 for tick control per year, which is out of reach of any LMIC household. In USA, a neighborhood of some 320 properties will need $64,000 for one tick control method, $162,000 for two control methods. At the animal health level: threshold treatment and production. Public funding is required for research, development and application of tick control interventions.	^94^
Limited success in control of tick loads using tick management programs	Despite efforts, the number of tick-borne disease cases have continued to rise in USA and Canada. New tick management programs with novel interventions are required.	^94^
Training	Public awareness on the need for tick control is lacking globally. Information on proper use of acaricides, acaricide resistance and vaccine acceptance (should vaccines become available) are essential and should be implemented.	^95^
Combination of control strategies is needed.	In the case of controlling *Ixodes* spp. ticks, evidence points towards the need to use multiple interventions simultaneously. This increases costs but rational use of strategies throughout seasons may reduce costs.	
Selection of acaricide for treatment and resistance diagnostics	Apart from bioassays, no rapid, standardized, and affordable diagnostics are available for acaricide resistance in *Hyalomma* vector ticks. Genome, transcriptome, and SNP identification are essential for rapid diagnostics. One cannot extrapolate from model tick species such as *R. microplus* for which data is available. Geographically focused SNPs have been shown to be involved in resistance development and analysis should be implemented for the characterization of sensitive tick populations.	
Tick rearing under laboratory conditions	Progress is made on rearing *Hyalomma* ticks in the laboratory. Future research will enable rapid screening of acaricides and transmission and/or transmission-blocking studies.	^96^
The role of co-infections	It is well described that co-infections alter host immunity and may affect vaccine protective capacity under field conditions. Co-infection in host animals with high *Hyalomma* tick loads are limited but molecular surveillance is required to evaluate its effect on vaccination.	^96^
Genomic data lacking for rational vaccine and control design	Genomes and transcriptome data for *Hyalomma* tick spp. are lacking and research with modern omics technologies is required to reduce errors on annotations currently present in public databases.	^97^
Surveillance and monitoring	Important to establish robust surveillance systems to monitor tick populations, acaricide resistance, track disease prevalence, and identify emerging hotspots to enable proactive control measures. This is costly and will require production of extensive and updated NGS datasets to design targeted assays and advance in this area.	
Import of ticks	Threats such as the importation of exotic ticks through human activity, combined with changes to either the habitat or climate could increase the risk of TBD appearance, persistence and transmission. New effective interventions and control strategies are required to reduce risks associated with new tick spp.	^98^
Bird migration	Monitoring and prevention of ticks introduced into new areas by bird migration is needed.	^98^
Stakeholder involvement	The involvement of multiple stakeholders, spanning from public health professionals and veterinarians to citizen scientists, is essential for effective tick control. Without their collective input, the implementation of mitigation strategies aimed at reducing pathogen transmission and impact becomes difficult. Moreover, the capacity to monitor the effects of climate change or landscape modifications on the risk of TBD becomes more challenging. However, like many interventions in public and animal health, it is necessary a cost-benefit assessment to determine most suitable and impactful interventions.	
Vector competence experiments for identification of disease hotspots	Many parasitic, viral, and bacterial pathogens are transmitted by ticks from different *Hyalomma* spp. However, limited vector competence experiments have been implemented, primarily attributed to challenges associated with executing a full cycle in controlled experimental settings. Facing these challenges requires “healthy” tick colonies, vertebrate hosts adapted to both ticks and pathogens, implementation of effective artificial tick feeding techniques, availability of pathogen cultures, and the provision of high biosafety level facilities, among other factors.	^53,86^

## Quality of life

To better implement an effective and efficacious CCHF control, it is important to consider healthcare system improvement, disease transmission prevention, and improved disease outcome. Risk factors include percutaneous exposure, mucosal contact with blood or sanguineous body fluids, intact skin contact with blood or other body fluids, physical contact with a CCHF patient without wearing gloves, and proximity to CCHF patients closer than one meter without a mask.

Human cases of CCHF most frequently occur among agricultural and livestock workers and veterinarians, transmitted by tick bites or infected animal contact. Healthcare workers (HCWs) are second only to farmers as a risk group^56^. Nosocomial cases are uncommon but cannot be ignored considering the high incidence rate (49%) and up to 66% case fatality rate^56,57^. Therefore, it is important to analyze the potential nosocomial transmission route and provide optional improvement within healthcare settings.

Risk factors include percutaneous exposure, mucosal contact with blood or sanguineous body fluids, intact skin contact with blood or other body fluids, physical contact with a CCHF patient without wearing gloves, and proximity to CCHF patients closer than one meter without a mask^58,59^. Availability of appropriate protective equipment and education of HCWs about safe clinical practices and infection control is the mainstay for the prevention of nosocomial spread of CCHF^59^. Established CCHF case management guidance, including suspected patient serology screening and isolation of CCHF cases in negative-pressure room are available.

To organize regular education programs within hospitals in endemic or high-risk areas are key interventions. Previous studies indicated that the awareness of the spread dynamic, clinical presentation, infection control and prevention of CCHF among HCWs in endemic countries is insufficient^60^. Subsequent to an hour lecture on infectious diseases, the knowledge level increased significantly^61^. One study^58^ investigated the compliance of HCWs during a follow-up visit to a hospital and found that the total seropositivity for CCHFV IgG was only 0.53%. It was assumed that this low rate may be related to high compliance with the use of personal protective equipment (PPE) and yearly education and awareness programs^58^. Thus, we recommend that regular education program in endemic countries should be provided and made compulsory to all related HCWs before the onset of the endemic season in endemic countries.

Developing occupational training programs is needed for HCWs in emergency and specialist departments to undergo training on recognizing potential CCHF cases and preventing disease transmission. This training should include consolidating potential exposure history, assessing occupation, and recognizing clinical symptoms indicative of a potential CCHF case as early as possible. Additionally, they should be educated on how to protect themselves while attending to suspected patients, which includes wearing PPE such as facial shields, goggles, gloves, and gowns. Additionally, prevention of needlestick injury should be included in the training. The training provided to laboratory technicians, housekeeping workers, and cleaners should prioritize the correct utilization of PPE, safe procedures for patient sample collection and handling, and effective disposal of medical waste.

Enhanced post-exposure prophylaxis, including molecular tests, serological tests, ribavirin, and supportive treatment, should be readily available and promptly accessible to HCWs at risk of exposure.

From policy perspective, there is a lack of an effective monitoring system for CCHF. We recommend the establishment of a global system for sharing case information and distinguishing high-risk areas. Given that high-risk populations predominantly reside in remote and developing areas, we strongly recommend that local governments proactively deliver regular health education on CCHF prevention and hazard recognition to these communities. This includes but is not limited to, providing education to butchers, abattoir workers, farmers, hunters, and other relevant groups residing in endemic areas^60^. Additionally, considering the high CFR of CCHF and living and economic conditions of high-risk populations, health insurance should offer coverage for CCHF-related treatment expenses. With this coverage, it would motivate patients to seek healthcare services and potentially contribute to preventing disease transmission to some extent.

## Outlook

The conclusions of the analysis for developing interventions for the control of CCHFV and tick vectors are summarized in Table 6. The most relevant gaps proposed are: (1) Vaccines with efficacy and effectiveness for the control of tick vector species and (2) Combining vaccines with other innovative control interventions, followed by (3) Effective and environmentally sustainable acaricides and repellents and (4) Evaluations of the integrated control interventions.

**Table 6:** Summary of main gaps and recommendations

Main gaps	Recommendations
Vaccines with efficacy and effectiveness for the control of multiple tick vector species	Chimeric antigens combining protective epitopes from different proteins. Vaccine formulation—understanding host immunity for informed decisions on adjuvant, route of administration and dose sparing. Post-translational modifications (e.g., alpha-Gal). Immunostimulants such as heat-inactivated mycobacteria with alpha-Gal for oral vaccine formulations. Personalized medicine with vaccine region, tick spp/strains, host approach. Different platforms for vaccine production and administration Optimal use of antigen production platforms for vaccine production and administration (global platforms are now available—e.g., Pirbright, UK, Design Biologix, SA).
Vaccines with efficacy and effectiveness for the control of CCHFV	Scalability and techno-economic analyses for production of antigens using selected vaccine platforms with proof of concept for production of lead candidates supported by in vivo studies. Coordinated regional/continental next-generation sequencing (RNAseq) efforts to identify lead antigens across species, allowing endeavors toward cross-protective vaccines. Determine if multivalent/polyvalent vaccines are needed for the most represented tick populations—for these analyses sufficient markers for population studies are required as ITS2 and COX does not allow for in-depth population genetics. Determine if inclusion of both viral glycoproteins and nucleocapsid are advantageous for optimal efficacy and control of virus spread (e.g., single injections—each able to elicit the immune response required for protection, vs combinatorial vaccines). Identify correlates of protection—these may not only be immunoglobulin but also T-cell responses.
Techno-economic analyses of tick and/or CCHF vaccine(s)	Regional uptake of a protective CCHF vaccine will be driven by demand, distribution infrastructure, and financial cost to humans.
Effective and environmentally sustainable acaricides and repellents	Growing acaricide resistance stresses to select a natural acaricide that will be suitable for control of tick population. For *Hyalomma* none has been described and validated. Evaluate tick susceptibility/resistance to available chemical acaricides (e.g., pyrethroids) and environmental impact. Establish rotational and selective protocols with acaricides to control multi-resistant tick populations. Screening of plant extracts to identify compounds for the production of effective and environmentally sound acaricides and tick repellents—combined with formulation optimization to enhance half-life on target species.
Combining vaccines with other innovative control interventions	Probiotics with the ability to act as immunostimulant to enhance vaccine protection and/or to overcome immune suppression during infection. Paratransgenesis to manipulate tick and host commensal bacteria. Target tick midgut microbiota to induce dysbacteriosis and reduce tick fitness. Gene-edited ticks (deletion of essential salivary gland protein(s)).
Evaluations of control interventions	Upscale of proof-of-concept lead candidates under controlled conditions that mimic the natural environment of the host (this is essential to ensure protection under conditions of co-infection) Evaluation of designed interventions in animal models (which are sub-optimal at this stage) and under field conditions in target host species. Address question, are wildlife vaccines required, practical or desirable? Evaluation of efficacy (pen-controlled conditions) and effectiveness (field trials) Consider cost-effectiveness, demand, and distribution infrastructure of designed interventions. Address the question, what is the economic model and value proposition for implementing anti-*Hyalomma* tick vaccines?
The role of host immunology in understanding tick-pathogen interaction	Considering tick-host-pathogens factors for designing vaccines and therapeutics. Establish exact mechanisms of the response to CCHFV and the role of specific proteins in inducing immunity.

## Supplementary information

Supplementary information
