# Supplementary material for: Interventions for the control of Crimean-Congo hemorrhagic fever and tick vectors
Source: NPJ Vaccines. 2024 Oct 1;9:181. doi: 10.1038/s41541-024-00970-5 (PMC11445411; doi:10.1038/s41541-024-00970-5)
Supplement: Supplementary file 1 — Supplementary information [file 41541_2024_970_MOESM1_ESM.pdf]

Supplementary information

Supplementary Table 1. CCHFV pre-clinical vaccines evaluated for efficacy in laboratory animals. Nucleic acid, subunit, vector-based, inactivated and other vaccines.

Vaccine	Animal species/strain	% Protection	Target(s)	Mechanism of protection	Clinical Trial?	References
**M-segment DNA vaccine**	IFNAR$^{-/-}$(C57BL/6), or C57BL/6 (MAR-5A3)	70% (25 µg dose) 100% (50 µg dose)	M-segment glycoproteins	$CD8^{+}$ T cells	No	1–3
**G_N/G_C and NP DNA vaccines**	IFNAR$^{-/-}$(A129), NHPs (DNA)	100% (50 µg dose mice) NHP (non-lethal model, no viremia, reduced vRNA in tissues, protects against mild disease)	G_N, G_C and NP	Unknown, In NHPs no neutralizing antibodies but T-cell response to GP in, NP response in NHPs is primarily humoral.	No	4–6
**S segment (NP) DNA +/- CD24**	IFNAR$^{-/-}$(A129) IFNAGR$^{-/-}$	100%	NP	unknown	No	7,8
**Bovine Herpesvirus NP subunit vaccine**	IFNAGR$^{-/-}$	100%	NP	unknown	No	9
**Adenovirus NP subunit vaccine**	IFNAR$^{-/-}$(C57BL/6)	33-78%	NP	prime/boost more protective	No	10
**mRNA+LNP G_N/G_C and/or NP**	IFNAR$^{-/-}$	100% (all forms)	M-segment glycoproteins and/or NP	unknown	No	11
**Replicating RNA NP (S) and GPC (M)**	C57BL/6 (MAR-5A3)	100% (NP alone protective, GPC alone not protective)	S and M-segment proteins	NP protective alone, GPC not protective alone	No	12a
	Rhesus macaque (*Macaca mulatta*)	Robust non-neutralizing humoral immunity had significant protection against the CCHFV challenge	S and M-segment proteins	Protective humoral response against NP	No	12b
**Chimpanzee adenoviral vector with M-segment (ChAdOx2 CCHF)**	IFNAR-/-(A129)	100	M-segment glycoproteins	antibody against glycoproteins, neutralizing antibody titers, and T-cell response but mechanism is unclear	Yes, Phase I currently recruiting (August 2023)	13

**Modified Vaccinia Ankara (MVA)-CCHF**	Humans	Ongoing	CCHFV	Humoral immune response	Yes, Phase I	https://www.hra.nhs.uk/planning-and-improving-research/application-summaries/research-summaries/phase-i-vaccine-study-of-mva-cchf/
**MVA-GP**	IFNAR-/-(A129)	100	M-segment glycoproteins	May require both cellular and humoral response	No	14,15
**rVSV expressing M-segment ORF**	STAT-1	100	M-segment glycoproteins	antibody against glycoproteins, and neutralizing antibody titers but mechanism is unclear	No	16
**CCHF virus-like replicon particle**	IFNAR-/-	103 TCID50 (80%), 105 TCID50 (100%)	M-segment glycoproteins and NP	May be primarily anti-NP antibodies	No	17–19
**Rhabdoviral-vector GP38 +/- GC**	C57BL/6 (MAR-5A3)	100	GP38 +/- GC	Likely non-neutralizing antibody, GC not needed	No	20
**Formalin inactivated cell culture derived CCHFV mixed with alum**	IFNAR-/-	5 µg dose (60%), 20 and 40 µg (80%)	Whole virus	antibody against glycoproteins, and neutralizing antibody titers but mechanism is unclear	No	21
**Mouse brain-derived chloroform and heat inactivated CCHFV adsorbed on Al(OH)3**	Humans	Unknown	Whole virus	antibody against GN/GC and N, T-cell response to N but mechanism is unclear	No, only used in Bulgaria	22

Supplementary Table 2. The *in vitro* efficacy of different extracts/EOs prepared from different plant parts against different species of *Hyalomma* ticks.

Name and part of plant	*In vitro* efficacy against	References
H. anatolicum		
Cymbopogon winterianus, leaves	LC50 against larvae = 0.14 %	23
Guiera senegalensis (Combrataceae), leaves	The LC50 for inhibition of hatchability of larvae was 1.71 % and 0.508 % using petroleum ether (PE) and ethanolic extracts (EE), respectively. The LC50 and LC99 against Larvae = 2.08 and 14.09 % using PE and 0.787 and 11.054 % using EE	24
Vitex negundo, extracts of leaves and roots	Against larvae the LC50 of root extract was 1.27 % and 0.011 % using leafextract	23
Withania somnifera, leaves	Against larvae the LC50 was 0.12 %	23
H. dromedarii		
Artemisia herba alba Asso (Asteraceae), aerial parts	Against larvae LC50 = 0.0022 to 0.369 µg/µl of different solvent guided extracts	25
A. monosperma Del. (Tarragon), Aerial parts	LC50 against larvae = 0.0437 µg/µl to 0.252 µg/µl of different solvent guided extracts	25
A. indica A Juss (Meliaceae), Neem oil and azadirachtin essential oil formulation	2.5 µg/ml	26
Euphorbia aegyptiaca (Euphorbiaceae), aerial parts	Against larvae LC50 ¼ 0.2595 µg/µl (DE), 1.511 µg/µl (EA), 0.763 µg/µl (hexane) and 0.6117 µg/µl (ethanol)	25
Francoeuria crispa (Asteraceae), Aerial parts	The LC50 against larvae in the range of 0.455 µg/µl to 1.069 µg/µl of extracts prepared using different solvents	25
Haplophyllumtuberculatum (Rutaceae), EOs from aerial parts	The LC50 against larvae = 0.5%	25
Mesembryanthemus forsskale (Aizoaceae), extracts of aerial parts	The LC50 against larvae using different solvent guided extracts was in the range of 0.611 µg/µl to 1.646 µg/µl	25
Reaumuria hirtella (Tamaricaceae), extracts of aerial parts	Against larvae The LC50 against larvae was in the range of 8.382 µg/µl to 124.68 µg/µl using different solvent guided extracts.	25
H. aegyptium		
A. herba alba (Asteraceae), aerialparts	LC50 = 1.105 against eggs, 0.755 against Larvae, and 0.0079 µL/ml against nymphs	27
H. scupense		
Eucalyptus camaldulensis Dehnh (river red gum), EOs from leaves and flowering tops	100 % inhibition of reproduction of female at 6.250 µl/ml; LC50 and LC95 against larvae = 0.207 µl/ml, 2.978 µl/ml, respectively	28
E. globules Labill (blue gum) EOs from leaves and flowering tops	100 % inhibition of the reproduction of female at 6.250 µl/ml; The LC50 and LC95 against larvae = 0.155 µl/ml and 5.183 µl/ml, respectively	28
Lavandula stoechas L. (lavender), The EOs from leaves and flowering tops	100 % inhibition of reproduction at 3.125 µl/ml. The LC50 and LC95 against larvae = 0.253 and 4.092µl/ml	28
Origanum floribundum Munby (oregano), The EOs from leaves and flowering tops	100 % inhibition of reproduction at 3.125 µl/ml. Against larvae LC50 and LC95 are 0.131 and 1.740 µl/ml, respectively	28
Rosmarinus officinalis L. (rosemary), The EOs from leaves and flowering tops	100 % inhibition of reproduction at 0.781 µl/ml. Against larvae the LC50 and LC95 values are 0.108 and 0.761 µl/ml, respectively	28

Thymus capitatus L. (thyme), The EOs from leaves and flowering tops.	100 % inhibit the reproduction in females at 1.562 µl/ml. Against larvae LC50 ¼ 0.058 µl/ml, LC90 ¼ 0.358 µl/ml, and LC95 ¼ 0.600 µl/ml	28
H. excavatum		
Azadirachta indica (Meliaceae), NeemAzal F (Commercial product prepared from seed)	LC50 = 1.0 % against newly hatched larvae, 0.5 % against unfed larvae and 1.6–3.2 % against unfed adults	29
H. rufipes		
E. globoidea, whole plant	repellent effects against adults (30–40 % of extract up to 120 min)	30
Nicotiana tabacum, whole plant extracts	Significant repellent effects on adult tick (40% v/w of extract for the first 40 min)	30
Senna italica subsp. *arachoides*(Fabaceae), root	Against adults the LC50 value is 8.66 % (w/v) in 24 h and 3.59 % (w/v) in 48 h	31
Tagetes minuta L. (Asteraceae), the EOs from aerial parts and flowers	Repellent EC50 for the repellent activity of male ticks is 0.072 ml/ml and for female ¼ 0.070 ml/ml	32
H. lusitanicum		
Geranium macrorrhizum (Geraniaceae), EOs from the aerial part	Against larvae LC50 ¼ 1.37 mg/ml and LC90 ¼ 2.87 mg/ml	33
H. marginatum		
Satureja thymbra L. (Lamiaceae), The EOs from aerial part of plant at flowering stage	100% mortality of adults in 3 h at 40 µl/l	34

Supplementary Table 3. Anti-*Hyalomma* spp. vaccine candidates.

Antigen(s)	Tick species	Characteristic	Stage of development	Reference
**Bm86 orthologs**				
Hd86	*H. scupense (detritum)*	Mid-gut protein<br>Bm86 orthologue	Control of *H. scupense* and *H. excavatum* rBm86 and rHd86 adjuvanted with Montanide 888; i.m. x3. Challenged with *H. scupense* larvae,<br>Hd86 caused 60% reduction of engorged *H. scupense* nymphs; Bm86 was ineffective. Hd86 was inefficient at controlling adult *H. scupense* ticks.	35
Haa86	*H. anatolicum*	Mid-gut protein<br>Bm86 orthologue	Experimental immunization of cross-bred calves with rHaa86 saponin in mineral oil was used to immunize, i.m. x3, challenged with *H. anatolicum* adults and larvae. 61.6% efficacy against challenge with adult *H. aanatolicum.*	36,37
Haa86	*H. anatolicum*	Mid-gut protein<br>Bm86 orthologue	Animal trial in cattle showing cross-protection to tick infestations and *Theileria annulata.*	38
Bm86	*H. anatolicum*	Mid-gut protein<br>Bm86 from *Rhipicephalus microplus*	Animal trial in cattle showing limited vaccine cross-protection (E = 25.1% and 44.5% for *H. anatolicum* and *R. microplus*, respectively). Species-specific antigens are required.	39,40
ATAQ	All hard ticks from *Metastriata* grp, including *Hyalomma*	Bm86 paralogue; present in gut and Malpighian tubules. May play role in cell growth and differentiation	*In silico*<br>Not yet investigated *in vitro* or *in vivo*.	41
**Subolesin (SUB)-derived antigens**				
Subolesin (SUB)	*H. anatolicum*	Intracellular protein. *Function:* transcription factor in the regulation of gene expression, affecting multiple cellular processes including the innate immune response, digestion, reproduction and development	Field tests<br>Recombinant Ha-SUB protein with Montanide ISA 50V2 used to immunize cross-bred calves i.m x3, then challenged with tick larvae (*R microplus* or *H anatolicum)*<br>Overall efficacy 65.4% (*H anatolicum)* and 54% (*R microplus).*	42
Q38 Subolesin/Akirin chimera containing conserved protective epitopes	*H. marginatum, H. lusitanicum*	Subolesin/Akirin regulatory proteins. Highly conserved across tick spp.	Efficacy of Q38 for the control of tick infestations in European roe deer (*Capreolus capreolus*)	43
Highly immunodominant epitopes of tick	*H.anatolicum* & CCHFV	Subolesin regulatory protein combined with	The designed vaccine was *in silico* validated for its physiochemical	44

Subolesin and major structural proteins (Nucleoprotein and Glycoprotein complex) of CCHFV		CCHFV structural proteins	properties, allergenicity and immunogenicity	
**Cathepsin L-like cysteine protease (CPL) orthologs**				
Cathepsin-L (CathL)	*H. anatolicum*	Digestive enzyme involved in haemoglobinolytic pathways	Field tests<br>Recombinant Ha-CathL protein with Montanide ISA 50V2 used to immunize cross-bred calves i.m x3, then challenged with tick larvae (*R microplus*or, *H. anatolicum)*<br>Overall efficacy 30.2% (*H anatolicum)* and 22.21% (*R microplus).*	42
Cathepsin L-like cysteine protease (CPL)<br>(Han CPL and HasCPL)	*H. anatolicum*<br>*H. asiaticum*	Digestive enzyme; Haemoglobinase produced in tick gut, salivary glands, ovaries & malpighian tubules	Pre-clinical study<br>rHasCPL or rHanCPL emulsified with Imject Alum adjuvant used to immunize rabbits s.c. x3, then rabbits were challenged with H. anatolicum adults.<br>Overall efficacy of HasCPL against heterologous tick challenge 54.8%	45,46
Cathepsin L-like cysteine protease (CPL)<br>(Han CPL and HasCPL)	*H. asiaticum*	Digestive enzyme; Haemoglobinase produced in tick gut, salivary glands, ovaries &malpighian tubules	Pre-clinical study<br>rHasCPL with interferon gamma as adjuvant. The protected rate of immunized mice from tick challenge was significantly higher after immunization with CPL + IFN-γ (85.11 %) than with CPL (63.28 %)	47
**Other tick-derived recombinant antigens**				
Ferritin 2 (FER2)	*H. anatolicum*	Secreted form of iron-binding protein expressed at all tick stages. major role in immune response, oxidative stress, blood acquisition and reproduction	Field tests<br>rHaFer2 with Montanide ISA 50V2 used to immunize cross-bred calves i.m. x3, then challenged with *H anatolicum* larvae or adults.<br>Protective efficacy of rHaFER2 against*H anatolicum* larvae - 51.8%; against adults - 51.2%	48
Tropomyosin (TPM)	*H. anatolicum*	actin associated salivary protein, regulates actin organization.	Field tests<br>rHaTPM with Montanide ISA 50V2 used to immunize cross-bred calves i.m. x3, then challenged with *H anatolicum* larvae or adults.<br>Protective efficacy of rHaTPM against *H anatolicum* larvae – 63.77%; against adults – 66.4%	48
Kunitz/bovine pancreatic trypsin inhibitor protein; HA11	*H. asiaticum*	Tick salivary gland protein; role in blood feeding including anticoagulant activity and disrupting host angiogenesis.<br>Most highly expressed in larval and nymphal stages	RNA interference & pre-clinical testing<br>HA11 gene disruption reduced tick feeding efficiency.<br>*H asiaticum*ticks feeding on rabbits immunized with HA11 had reduced engorged body weight	49
Calreticulin (CRT)	*H. anatolicum*	Calcium binding protein.<br>Function: both extra-cellular and intra-cellular functions, involved in evading the host's immune system in ticks	Field tests<br>Recombinant Ha-CRT protein with Montanide ISA 50V2 used to immunize cross-bred calves i.m x3, then challenged with tick larvae (*R microplus*or*H anatolicum)*<br>Overall efficacy 41.3% (*H anatolicum)* and 37.56% (*R microplus).*	42
**Tick protein extracts/fractions**				
Cement-coneproteins (23 kDaprotein)	*H. anatolicum*<br>*H. aegyptium*	Proteins part of tick cement cone	Preliminary evaluation in cattle	50
Cross-reactive protein fraction from the adults of the hard tick *H. dromedarii* isolated by Cyanogen	*H.dromedarii*	Unknown	Preliminary evaluation in rabbits	51

Bromide-activated Sepharose-4B affinity column chromatography				
Three major glycoproteins (GLPs; 97, 66 and 40 kDa) purified from adult and larvae	*H. dromedarii*	Adults and larvae	Preliminary evaluation in rabbits	52
A 34 k Da glycoprotein with saponin was used for immunization	*H. anatolicum*	Larvae	A 56% and 52.44 protection against challenged larvae and adults, respectively was noted	53
The affinity purified soluble antigen of 37KDa (GHLAgP)	*H. anatolicum*	Larvae	Cross-bred male calves were immunized with GHLAgP and infected with sub-lethal dosages of *Theileria annulata*. A significant protection against challenged adults and larvae were noted a significant decrease in *T. annulata* infection rate in ticks fed on immunized cattle in comparison to control was noted	54
Two glycoproteins of 34 and 29 kDa were isolated from *H. anatolicum* and *R. microplus*, respectively employing two steps affinity chromatography	*H. anatolicum* *R. microplus*	Larvae	Immunization of crossbred male calves using the isolated glycoproteins together conferred a Protection level of 73.6% and 75.0% of challenged larvae and adults of *H. anatolicum* and 89.8% adults of *R. microplus*.	55
Soluble nymphal 39 kDa antigen (HNAg) purified by immunoaffinity chromatography using CNBr-activated Sepharose 4B coupled with immunoglobulin ligands from animals immunized with HNAg	*H. anatolicum*	Nymphs	Following immunization of crossbred calves with HNAg in three doses, significant rejections of larvae ($p<0.001$, 84.2%), nymphs ($p<0.05$, 61.4%) and adults ($p<0.05$, 58.7%) were recorded	56
Soluble gut specific larval antigens were purified by immunoaffinity chromatography using anti-gut IgG as ligand. The antigens, Aff-GHLAg, having the molecular weight of 100, 59.4 and 37 kDa	*H. anatolicum*	Larvae	The Aff-GHLAg was used to immunize 6–7 months old cross-bred (*Bos taurus* &*B. indicus*) calves. In three dosages with FCA and IFA. A protection level of 70.6%, 54.5% and 61.9% was recorded against larvae, nymphs and Adults, respectively	57
A 39kDa antigens was purified by immunoaffinity chromatography using immunoglobulin ligands from cross-bred animals immunized with soluble larval antigen. The Affinity-purified antigen (A-TLE) and a total larval extract (TLE) were used to immunize cross-bred (*Bos indicus* x *B. taurus*) cattle	*H. anatolicum*	larvae	The group immunized with Aff-TLE rejected71.6% of larvae and 77.3% of nymphs. However, the rejection percentages were lower in the TLE-immunized group. A significant decrease in the number of resultant nymphs ($p < 0.01$) and adults ($p< 0.01$) in the ticks fed on the A¡-TLE-immunized group	58

Soluble larval antigen with FCA was used to immunize calves	*H. anatolicum*	larvae	A protection level of 57.25 + 6.8% and 45.75 + 5.16% against challenged larvae and nymphs, respectively was noted	59
**Multiepitope peptides as immunogen**				
Two multi-epitopic peptides (MEPs), targeting Ferritin 2, tropomyosin and vitellogenin receptor genes were designed.	*H. anatolicum*	Unknown	Rabbits were immunized by MEPs mixed with 8% Montanide TM gel 01PR. Following challenge, a 93.3% to 96.9% protection against challenged larvae and 86.4 to 89.9% against adults was noted	60

Supplementary Table 4. Compendium of some of the most widely used strategies for suppressing tick populations in rodents.

Intervention / Cost estimates	Advantages / Limitations	References
Landscaping and vegetation management	Non-chemical control<br><br>Scaling up beyond individual backyards to communities will require administration, funding and oversight.<br><br>Impact on green spaces and public lands<br><br>Importance of management around schools<br><br>Can be cost effective if xeric barriers are used (e.g., wood chips, gravel, river stone).<br><br>Pasture spelling/burning, wherever possible.	61,62
Broad-cast application of synthetic, natural and fungal acaricides	Reluctance to use effective chemical control agents.<br><br>Cost for companies/government employees to apply compounds – USA average is U$172/h.<br><br>Product cost for natural and fungal acaricides exceed those of synthetics paying for the "green" option.<br><br>Natural acaricides require more frequent application.<br><br>Liquid and granular formulations of acaricides for rodents/small animals. High cost: Cyhalthrin (pyrethroid) is some $64/ha.<br><br>Sprayers and spreaders are impractical for treating larger areas of tick habitat with limited availability and often not stable under field conditions.	62
Deployment of host-targeted acaricides	Tick Tubes and Thermacell Tick Control Tubes. Tick tubes filled with cotton balls treated with 7.4% permethrin.<br><br>Examples sold commercially include Damminix.<br><br>Which acaricide to use in acaricide resistant areas?<br><br>Size needed for optimal tick control? Safety. Half-life of products under environmental conditions.<br><br>Rodent Bait boxes: These are child resistant plastic boxes with a bait attractant and fipronil-treated felt wick enabling passive treatment. Commercial products include SELECT TCS and TICK BOX TCS rodent bait boxes. Placing of these require a pesticide applicator license in some countries.<br><br>Seasonal application can reduce costs.	63–65
Reduction of host animal species	Hare and rodent populations can be controlled. A number of rodent control strategies have been described.<br><br>Wildlife populations is more challenging and may be under conservation regulation. Permit may be required.<br><br>For some larger animal hosts, something similar to the A 4-Poster deer treatment station may be envisaged. These are feed stations that uses corn to bait deer and treat the deer with a pesticide to kill ticks. A deer rubs against rollers containing an acaricide, a pesticide specially formulated to kill ticks and mites, as it lowers its head to the trough to feed on the corn at the station.	66

Geographic information system and mapping	Essential to identify regions for priority control.<br><br>Lacking in most countries for *Hyalomma* and CCHFV.	
Oral vaccine and acaricide	Lessons learned from control of *I. scapularis* and *I. pacificus* will be essential in the development of oral vaccines in rodents. Vaccines targeting viruses has not been commercialized. However, a vaccine against *Borrelia* is expected to be commercialized in the next year.<br><br>An oral acaricide for rodents is anticipated to be commercialized in the next 2 years by Genesis Laboratories, Inc. (Wellington, CO, USA).	66

Supplementary Table 5. Strategies currently used to reduce human-mature tick contact.

Pasture management if possible	Most examples regarding tick control via pasture management are for *Rhipicephalus* ticks.<br><br>Rotational Grazing: Regularly rotate cattle between pastures to disrupt the life cycle of ticks and reduce their exposure to infested areas.<br><br>Mowing and Burning: Maintain a well-managed pasture by mowing tall grass and periodically burning areas to reduce tick habitat.	67
Maintenance of hosts naturally resistant to ticks	Various studies on cattle breeds resistant to ticks are published. None are specific to *Hyalomma* ticks. A recent GWAS study is provided as reference.	68
Pheromone impregnated decoys for attracting and killing ticks	The behaviors of interest are predominantly regulated by purines, substituted phenols, or cholesteryl esters, along with other pheromonal compounds such as organic acids, hematin, or ecdysteroids. Innovatively, devices have been created to merge these specific compounds, constituting pheromones, with an acaricide. When these devices are applied to vegetation infested with ticks or directly to the body surfaces of livestock or companion animals, they prove effective in controlling tick populations.	69
Biological control using natural enemies	Predatory Organisms: Introduce natural predators of ticks, such as certain species of birds and beneficial insects, to the cattle environment.<br><br>Entomopathogenic nematodes that parasitize and kill tick larvae and nymphs in the soil.	
Diatomaceous Earth	The inert dusts kaolin, silica gel, perlite, and diatomaceous earth are lethal to ixodids.	70
Social education and communication	Education - communication for reduced risk of contact/transmission	

Supplementary Panel 1. Evolution of anti-*Hyalomma* spp. vaccine protective antigens.

- The attempt to identify candidate antigens against *Hyalomma* spp. was initiated with experimental immunization of animals with stage specific antigens. For example, Ghosh et al. [71] immunized rabbits with extracts of larvae and nymphs and a significant reduction in the engorgement percentage, engorgement weight and egg masses in ticks fed on immunized animals compared to ticks fed on a control group of animals was recorded.
- Further, a 39 kDa larval and nymphal antigens were used for immunization and challenge study and a cumulative protection of 58% to more than 80% was recorded against larvae, nymphs, and adults of *H. anatolicum* [56,58].
- Then, several immunization trials using purified antigens were conducted and achieved encouraging protection against *H. anatolicum* challenge [55,57,72]. El Hakim et al. [52] evaluated three major glycoproteins (97, 66 and 40 kDa) purified from *H. dromedarii* adults and larvae and demonstrated 63–67 % reduction in egg hatchability of ticks fed on immunized animals in comparison to ticks fed on control animals.
- Subsequently, de Vos et al. [73] identified a homologue of Bm86 in *H. anatolicum* with 50% protection efficacy. Ben Said et al. [74] cloned and characterized the Hd86 antigen from *H. scupense*, an ortholog of the Bm86 gene and reported very low intra-specific diversity in amino acid sequences of Hd86 of different isolates of *H. scupense* of Tunisia and a 59.1 % protection against the nymphal stage of *H. scupense* and no protection against adults was reported [35] and reduction of Hd86 gene transcript in adults is identified as the possible reasons of the variation in results. Concurrently, Ben Said et al. [75] studied Bm86 ortholog in four different *Hyalomma* spp. such as *H. marginatum* (Hm86), *H. excavatum* (He86), *H. dromedarii* (Hdr86) and *H. scupense* (Hd86-A1) and suggested that Hd86-A1 vaccine candidate might be more appropriate to target *Hyalomma* tick species in contrast to Bm86 commercial vaccines.
- Further, Haa86, a Bm86 homologue of *H. anatolicum* was tested against homologous challenge infestations and a protection level of 47-60% and 40-80% against larvae and adults, respectively was noted [36–39]. Subsequently, Subolesin (SUB), Calreticulin (CRT), Cathepsin-L like cysteine protease (CathL), Ferritin 2 (FER2) and Tropomyosin (TPM) ortholog of *H. anatolicum* were tested and 65.4%, 63.7%, 51.7%, 41.3% and 30.2%, respectively [42,48].

Supplementary Figure 1. Bibliometric analysis of CCHF vaccines.

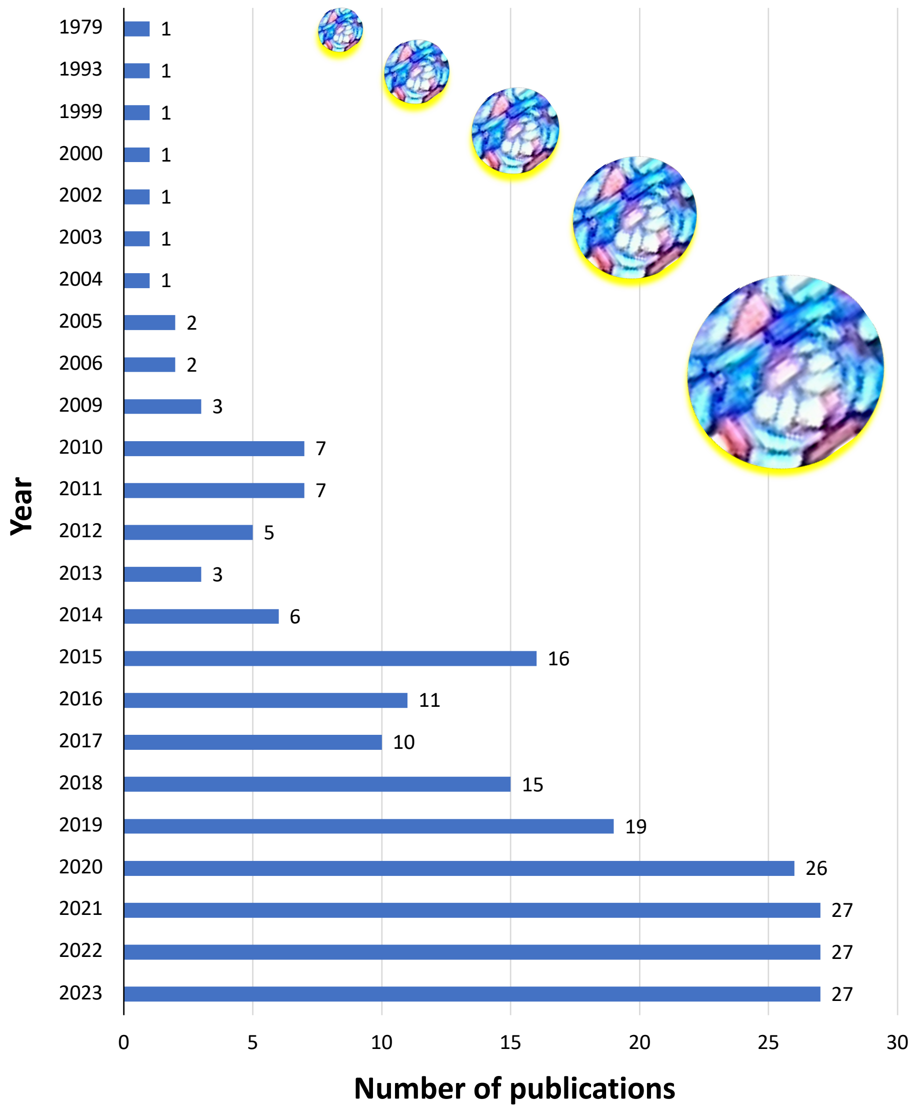

Number of publications in PubMed (https://pubmed.ncbi.nlm.nih.gov) searching with terms Crimean AND Congo AND Hemorrhagic AND Fever AND Vaccine on November 2, 2023.

References

1 Garrison AR, Shoemaker CJ, Golden JW, *et al.* A DNA vaccine for Crimean-Congo hemorrhagic fever protects against disease and death in two lethal mouse models. *PLoS Negl Trop Dis* 2017; **11**: e0005908.

2 Suschak JJ, Golden JW, Fitzpatrick CJ, *et al.* A CCHFV DNA vaccine protects against heterologous challenge and establishes GP38 as immunorelevant in mice. *Npj Vaccines* 2021; **6**: 1–11.

3 Golden JW, Fitzpatrick CJ, Suschak JJ, *et al.* Induced protection from a CCHFV-M DNA vaccine requires CD8+ T cells. *Virus Research* 2023; **334**: 199173.

4 Hinkula J, Devignot S, Åkerström S, *et al.* Immunization with DNA plasmids coding for Crimean-Congo hemorrhagic fever virus capsid and envelope proteins and/or virus-like particles induces protection and survival in challenged mice. *J Virol* 2017; **91**: e02076-16.

5 Hawman DW, Ahlén G, Appelberg KS, *et al.* A DNA-based vaccine protects against Crimean-Congo haemorrhagic fever virus disease in a *Cynomolgus macaque* model. *Nat Microbiol* 2021; **6**: 187–95.

6 Hawman DW, Meade-White K, Leventhal S, *et al.* Accelerated DNA vaccine regimen provides protection against Crimean-Congo hemorrhagic fever virus challenge in a macaque model. *Mol Ther* 2023; **31**: 387–97.

7 Aligholipour Farzani T, Földes K, Ergünay K, Gurdal H, Bastug A, Ozkul A. Immunological analysis of a CCHFV mRNA vaccine candidate in mouse models. *Vaccines (Basel)* 2019a; **7**: 115.

8 Aligholipour Farzani T, Földes K, Hanifehnezhad A, *et al.* Bovine Herpesvirus Type 4 (BoHV-4) vector delivering nucleocapsid protein of Crimean-Congo hemorrhagic fever virus induces comparable protective immunity against lethal challenge in IFNα/β/γR-/- mice models. *Viruses* 2019b; **11**: 237.

9 Aligholipour Farzani T, Hanifehnezhad A, Földes K, *et al.* Co-delivery effect of CD24 on the immunogenicity and lethal challenge protection of a DNA vector expressing nucleocapsid protein of Crimean Congo hemorrhagic fever virus. *Viruses* 2019c; **11**: 75.

10 Zivcec M, Safronetz D, Scott DP, Robertson S, Feldmann H. Nucleocapsid protein-based vaccine provides protection in mice against lethal Crimean-Congo hemorrhagic fever virus challenge. *PLoS Negl Trop Dis* 2018; **12**: e0006628.

11 Appelberg S, John L, Pardi N, *et al.* Nucleoside-modified mRNA vaccines protect IFNAR-/- mice against Crimean-Congo hemorrhagic fever virus infection. *J Virol* 2022; **96**: e0156821.

12a Leventhal SS, Meade-White K, Rao D, *et al.* Replicating RNA vaccination elicits an unexpected immune response that efficiently protects mice against lethal Crimean-Congo hemorrhagic fever virus challenge. *EBioMedicine* 2022; **82**: 104188.

12b Hawman DW, Leventhal SS, Meade-White K, et al. A replicating RNA vaccine confers protection in a rhesus macaque model of Crimean-Congo hemorrhagic fever. *NPJ Vaccines* 2024; **9**: 86.

13 Saunders JE, Gilbride C, Dowall S, *et al.* Adenoviral vectored vaccination protects against Crimean-Congo haemorrhagic fever disease in a lethal challenge model. *eBioMedicine* 2023; **90**: 104523.

14 Buttigieg KR, Dowall SD, Findlay-Wilson S, *et al.* A Novel Vaccine against Crimean-Congo haemorrhagic fever protects 100% of animals against lethal challenge in a mouse model. *PLOS ONE* 2014; **9**: e91516.

15 Dowall SD, Graham VA, Rayner E, *et al.* Protective effects of a Modified Vaccinia Ankara-based vaccine candidate against Crimean-Congo haemorrhagic fever virus require both cellular and humoral responses. *PLOS ONE* 2016; **11**: e0156637.

16 Rodriguez SE, Cross RW, Fenton KA, Bente DA, Mire CE, Geisbert TW. Vesicular stomatitis virus-based vaccine protects mice against Crimean-Congo hemorrhagic fever. *Sci Rep* 2019; **9**: 7755.

17 Scholte FEM, Spengler JR, Welch SR, *et al.* Single-dose replicon particle vaccine provides complete protection against Crimean-Congo hemorrhagic fever virus in mice. *Emerg Microbes Infect* 2019; **8**: 575–8.

18 Spengler JR, Welch SR, Scholte FEM, *et al.* Heterologous protection against Crimean-Congo hemorrhagic fever in mice after a single dose of replicon particle vaccine. *Antiviral Research* 2019; **170**: 104573.

19 Scholte FEM, Karaaslan E, O'Neal TJ, *et al.* Vaccination with the Crimean-Congo hemorrhagic fever virus viral replicon vaccine induces NP-based T-cell activation and antibodies possessing Fc-mediated effector functions. *Front Cell Infect Microbiol* 2023; **13**: 1233148.

20 Scher G, Bente DA, Mears MC, Cajimat MNB, Schnell MJ. GP38 as a vaccine target for Crimean-Congo hemorrhagic fever virus. *NPJ Vaccines* 2023; **8**: 73.

21 Canakoglu N, Berber E, Tonbak S, *et al.* Immunization of knock-out α/β interferon receptor mice against high lethal dose of Crimean-Congo hemorrhagic fever virus with a cell culture-based vaccine. *PLOS Neglected Tropical Diseases* 2015; **9**: e0003579.

22 Mousavi-Jazi M, Karlberg H, Papa A, Christova I, Mirazimi A. Healthy individuals' immune response to the Bulgarian Crimean-Congo hemorrhagic fever virus vaccine. *Vaccine* 2012; **30**: 6225–9.

23 Singh NK, Rath SS. Esterase mediated resistance against synthetic pyrethroids in field populations of *Rhipicephalus (Boophilus) microplus* (Acari: Ixodidae) in Punjab districts of India. *Vet Parasitol* 2014; **204**: 330–8.

24 Osman IM, Mohammed AS, Abdalla AB. Acaricidal properties of two extracts from *Guiera senegalensis* J.F. Gmel. (Combrataceae) against *Hyalomma anatolicum* (Acari: Ixodidae). *Vet Parasitol* 2014; **199**: 201–5.

25 Abdel-Shafy S, Soliman MMM, Habeeb SM. In vitro acaricidal effect of some crude extracts and essential oils of wild plants against certain tick species. *Acarologia* 2007; **47**: 33–42.

26 Al-Rajhy DH, Alahmed AM, Hussein HI, Kheir SM. Acaricidal effects of cardiac glycosides, azadirachtin and neem oil against the camel tick, *Hyalomma dromedarii* (Acari: Ixodidae). *Pest Manag Sci* 2003; **59**: 1250–4.

27 Laghzaoui E-M, Ayoub K, Abbad A, Leach D, Spooner-Hart R, El Mouden EH. Acaricidal properties of essential oils from Moroccan plants against immature ticks of *Hyalomma aegyptium* (Linnaeus, 1758); an external parasite of the spur-thighed tortoise (*Testudo graeca*). *Int J Acarol* 2018; **44**: 1–7.

28 Djebir S, Ksouri S, Trigui M, *et al.* Chemical composition and acaricidal activity of the essential oils of some plant species of *Lamiaceae* and *Myrtaceae* against the vector of tropical bovine theileriosis: *Hyalomma scupense* (syn. *Hyalomma detritum*). *BioMed Research International* 2019; **2019**: e7805467.

29 Abdel-Shafy S, Zayed AA. In vitro acaricidal effect of plant extract of neem seed oil (*Azadirachta indica*) on egg, immature, and adult stages of *Hyalomma anatolicum* excavatum (Ixodoidea: Ixodidae). *Vet Parasitol* 2002; **106**: 89–96.

30 Magano S. Repellent properties of Nicotiana tabacum and Eucalyptus globoidea against adults of *Hyalomma marginatum rufipes*. *Afr J Microbiol Res* 2011; **5**: 4508-4512.

31 Magano SR, Thembo KM, Ndlovu SM, Makhubela NFH. The anti-tick properties of the root extracts of *Senna italica* subsp. *arachoides*. *Afr J Biotechnol* 2008; **7**: 476-481.

32 Nchu F, Magano SR, Eloff JN. In vitro anti-tick properties of the essential oil of *Tagetes minuta L.* (Asteraceae) on *Hyalomma rufipes* (Acari: Ixodidae). *Onderstepoort J Vet Res* 2012; **79**: E1-5.

33 Navarro-Rocha J, F. Barrero A, Burillo J, Olmeda AS, González-Coloma A. Valorization of essential oils from two populations (wild and commercial) of *Geranium macrorrhizum L. Ind Crops Prod* 2018; **116**: 41–5.

34 Cetin H, Cilek JE, Oz E, Aydin L, Deveci O, Yanikoglu A. Acaricidal activity of *Satureja thymbra* L. essential oil and its major components, carvacrol and gamma-terpinene against adult *Hyalomma marginatum* (Acari: Ixodidae). *Vet Parasitol* 2010; **170**: 287–90.

35 Galaï Y, Canales M, Ben Saïd M, *et al.* Efficacy of *Hyalomma scupense* (Hd86) antigen against *Hyalomma excavatum* and *H. scupense* tick infestations in cattle. *Vaccine* 2012; **30**: 7084–9.

36 Azhahianambi P, de la Fuente J, Suryanarayana VVS, Ghosh S. Cloning, expression and immunoprotective efficacy of rHaa86, the homologue of the Bm86 tick vaccine antigen, from *Hyalomma anatolicum anatolicum*. *Parasite Immunol* 2009; **31**: 111–22.

37 Azhahianambi P, Ray DD, Chaudhuri P, Gupta R, Ghosh S. Vaccine efficacy of Bm86 ortholog of *H. a. anatolicum*, rHaa86 expressed in prokaryotic expression system. *J Parasitol Res*. 2009; **2009**:165812.

38 Jeyabal L, Kumar B, Ray D, Azahahianambi P, Ghosh S. Vaccine potential of recombinant antigens of *Theileria annulata* and *Hyalomma anatolicum anatolicum* against vector and parasite. *Vet Parasitol* 2012; **188**: 231–8.

39 Kumar B, Murugan K, Ray DD, Ghosh S. Efficacy of rBm86 against *Rhipicephalus (Boophilus) microplus* (IVRI-I line) and *Hyalomma anatolicum anatolicum* (IVRI-II line) infestations on bovine calves. *Parasitol Res* 2012a; **111**: 629–35.

40 Kumar B, Azhahianambi P, Ray DD, *et al.* Comparative efficacy of rHaa86 and rBm86 against *Hyalomma anatolicum anatolicum* and *Rhipicephalus (Boophilus) microplus*. *Parasite Immunol* 2012b; **34**: 297–301.

41 Nijhof AM, Balk JA, Postigo M, Rhebergen AM, Taoufik A, Jongejan F. Bm86 homologues and novel ATAQ proteins with multiple epidermal growth factor (EGF)-like domains from hard and soft ticks. *Int J Parasitol* 2010; **40**: 1587–97.

42 Kumar B, Manjunathachar HV, Nagar G, *et al.* Functional characterization of candidate antigens of *Hyalomma anatolicum* and evaluation of its cross-protective efficacy against *Rhipicephalus microplus*. *Vaccine* 2017; **35**: 5682–92.

43 Contreras M, Kasaija PD, Kabi F, Mugerwa S, De la Fuente J. The correlation between Subolesin-reactive epitopes and vaccine efficacy. *Vaccines (Basel)* 2022; **10**: 1327.

44 Shrivastava N, Verma A, Dash PK. Identification of functional epitopes of structural proteins and in-silico designing of dual acting multiepitope anti-tick vaccine against emerging Crimean-Congo hemorrhagic fever virus. *Eur J Pharm Sci* 2020; **151**: 105396.

45 Song R, Zhai X, Fan X, *et al.* Prediction and validation of cross-protective candidate antigen of *Hyalomma asiaticum* cathepsin L between *H. asiaticum* and *H. anatolicum*. *Exp Appl Acarol* 2022; **86**: 283–98.

46 Song R, Ge T, Hu E, *et al.* Recombinant cysteine proteinase as anti-tick targeting *Hyalomma asiaticum* infestation. *Exp Parasitol* 2022; **235**: 108234.

47 Song R, Zhai X, Fan X, *et al.* Recombinant interferon-gamma promotes immunoglobulin G and cytokine memory responses to cathepsin L-like cysteine proteinase of *Hyalomma asiaticum* and the efficacy of anti-tick. *Vet Immunol Immunopathol* 2021; **235**: 110201.

48 Manjunathachar HV, Kumar B, Saravanan BC, *et al.* Identification and characterization of vaccine candidates against *Hyalomma anatolicum*—Vector of Crimean-Congo haemorrhagic fever virus. *Transbound Emerg Dis* 2019; **66**: 422–34.

49 Zhang H, Qiao R, Gong H, Cao J, Zhou Y, Zhou J. Identification and anticoagulant activity of a novel Kunitz-type protein HA11 from the salivary gland of the tick *Hyalomma asiaticum*. *Exp Appl Acarol* 2017; **71**: 71–85.

50 Rafiq N, Naseem M, Kakar A, Shirazi JH, Masood MI. A preliminary evaluation of tick cement-cone protein extract for a vaccine against Hyalomma infestation. *Iran J Vet Res* 2022; **23**: 255–64.

51 Toaleb NI, Gabr HSM, Abd El-Shafy S, Abdel-Rahman EH. Evaluation of vaccine candidates purified from the adult ticks of *Ornithodoros savignyi* (Acari: Argasidae) and *Hyalomma dromedarii* (Acari: Ixodidae) against tick infestations. *J Parasit Dis* 2019; **43**: 246–55.

52 El Hakim AE, Shahein YE, Abdel-Shafy S, Abouelella AMK, Hamed RR. Evaluation of glycoproteins purified from adult and larval camel ticks (*Hyalomma dromedarii*) as a candidate vaccine. *J Vet Sci* 2011; **12**: 243–9.

53 Ghosh S, Azhahianambi P, Ray DD, Gupta SC, Bansal GC, Choudhury P. Anti-tick effects sf 34kDa glycoprotein of *Hyalomma anatolicum anatolicum* in combination with adjuvant. *Indian J Anim Sci* 2008; **78**: 17-23.

54 Das G, Ghosh S, Ray DD. Reduction of *Theileria annulata* infection in ticks fed on calves immunized with purified larval antigens of *Hyalomma anatolicum anatolicum*. *Trop Anim Health Prod* 2005; **37**: 345–61.

55 Singh NK, Ghosh S. Experimental immunisation of crossbred cattle with glycoproteins isolated from the larvae of *Hyalomma anatolicum anatolicum* and *Boophilus microplus*. *Exp Appl Acarol* 2003; **31**: 297–314.

56 Sharma JK, Ghosh S, Khan MH, Das G. Immunoprotective efficacy of a purified 39 kDa nymphal antigen of *Hyalomma anatolicum anatolicum*. *Trop Anim Health Prod* 2001; **33**: 103–16.

57 Das G, Ghosh S, Khan MH, Sharma JK. Immunization of cross-bred cattle against *Hyalomma anatolicum anatolicum* by purified antigens. *Exp Appl Acarol* 2000; **24**: 645–59.

58 Ghosh S, Khan MH, Ahmed N. Cross-bred cattle protected against *Hyalomma anatolicum anatolicum* by larval antigens purified by immunoaffinity chromatography. *Trop Anim Health Prod* 1999; **31**: 263–73.

59 Ghosh S, Khan MH. Immunization of cattle against *Hyalomma anatolicum anatolicum* using larval antigens. *Indian J Exp Biol* 1999; **37**: 203–5.

60 Nandi A, Manisha, Solanki V, *et al.* protective efficacy of multiple epitope-based vaccine against *Hyalomma anatolicum*, vector of *Theileria annulata* and Crimean–Congo hemorrhagic fever virus. *Vaccines* 2023; **11**: 881.

61 Eisen L, Stafford KC. Barriers to effective tick management and tick-bite prevention in the United States (Acari: Ixodidae). *J Med Entomol* 2021; **58**: 1588–600.

62 Schulze TL, Eisen L, Russell K, Jordan RA. Community-based integrated tick management programs: cost and feasibility scenarios. *J Med Entomol* 2023; **60**: 1048–60.

63 Jordan RA, Schulze TL. Ability of Two Commercially Available Host-Targeted Technologies to Reduce Abundance of Ixodes scapularis (Acari: Ixodidae) in a Residential Landscape. *J Med Entomol* 2019; **56**: 1095–101.

64 Ostfeld RS, Mowry S, Bremer W, *et al.* Impacts over time of neighborhood-scale interventions to control ticks and tick-borne disease incidence. *Vector Borne Zoonotic Dis* 2023b; **23**: 89–105.

65 Ostfeld RS, Adish S, Mowry S, *et al.* Effects of Neighborhood-scale acaricidal treatments on infection prevalence of blacklegged ticks (*Ixodes scapularis*) with three zoonotic pathogens. *Pathogens* 2023a; **12**: 172.

66 Eisen L. Rodent-targeted approaches to reduce acarological risk of human exposure to pathogen-infected Ixodes ticks. *Ticks and Tick-borne Diseases* 2023; **14**: 102119.

67 Hüe T, Berger A, Wang H-H, Grant WE, Teel PD, de León AAP. Integrated control of the cattle tick, *Rhipicephalus australis* (Acari: Ixodidae), in New Caledonia through the Pasture and Cattle Management method. *Parasitol Res* 2021; **120**: 2749–58.

68 dos Santos CG, Sousa MF, Vieira JIG, *et al.* Candidate genes for tick resistance in cattle: a systematic review combining post-GWAS analyses with sequencing data. *Journal of Applied Animal Research* 2022; **50**: 460–70.

69 Sonenshine DE. Tick pheromones and their use in tick control. *Annu Rev Entomol* 2006; **51**: 557–80.

70 Showler AT, Saelao P. Integrative alternative tactics for Ixodid control. *Insects* 2022; **13**: 302.

71 Ghosh S, Khan MH, Gupta SC. Immunization of rabbits against *Hyalomma anatolicum anatolicum* using homogenates from unfed immature ticks. *Indian J Exp Biol*. 1998;36(2):167-70.

72 Das G, Ghosh S, Sharma JK, Khan MH, Gupta SC. Attempted immunization of crossbred (*Bos taurus* x *Bos indicus*) calves by affinity purified concealed antigens of *Hyalomma anatolicum anatolicum*. *Indian J Anim Sci*. 2003; **73**: 713-7.

73 de Vos S, Zeinstra L, Taoufik O, Willadsen P, Jongejan F. Evidence for the utility of the Bm86 antigen from *Boophilus microplus* in vaccination against other tick species. *Exp Appl Acarol*. 2001;**25**: 245-61.

74 Ben Said M, Galai Y, Canales M, et al. Hd86, the Bm86 tick protein ortholog in *Hyalomma scupense* (syn. *H. detritum*): expression in *Pichia pastoris* and analysis of nucleotides and amino acids sequences variations prior to vaccination trials. *Vet Parasitol*. 2012a;183: 215-23.

75 Ben Said M, Galai Y, Mhadhbi M, Jedidi M, de la Fuente J, Darghouth MA. Molecular characterization of Bm86 gene orthologs from *Hyalomma excavatum*, *Hyalomma dromedarii* and *Hyalomma marginatum marginatum* and comparison with a vaccine candidate from *Hyalomma scupense*. *Vet Parasitol*. 2012b;190: 230-40.
